# Disentangling the genetic underpinnings of neuropsychiatric symptoms in Alzheimer’s Disease in the Alzheimer’s Disease Sequencing Project: study design and methodology

**DOI:** 10.1002/alz.092344

**Published:** 2025-01-03

**Authors:** Ajneesh Kumar, Nicholas R. Ray, Andrew F Zaman, Masood Manoochehri, Colin Stein, Pedro R. Mena, Robert Sweet, Timothy J. Hohman, Michael L. Cuccaro, Gary W Beecham, Edward D. Huey, Christiane Reitz

**Affiliations:** ^1^ Columbia University, New York, NY USA; ^2^ Columbia University Irving Medical Center, New York, NY USA; ^3^ John P. Hussman Institute for Human Genomics, University of Miami Miller School of Medicine, Miami, FL USA; ^4^ Brown University, Providence, RI USA; ^5^ Department of Psychiatry and Neurology, University of Pittsburgh, Pittsburgh, PA USA; ^6^ Vanderbilt Memory and Alzheimer’s Center, Vanderbilt University Medical Center, Nashville, TN USA; ^7^ John P. Hussman Institute for Human Genomics, Miller School of Medicine, Miami, FL USA; ^8^ G. H. Sergievsky Center, Vagelos College of Physicians and Surgeons, Columbia University, New York, NY USA

## Abstract

**Background:**

Neuropsychiatric Symptoms (NPS) including aggression, psychosis, anxiety, apathy and depression are highly prevalent in Alzheimer’s Disease patients and are associated with accelerated decline and a detrimental impact on suffering and quality of life of both patients and caregivers. There are no effective pharmaceutical interventions targeting these symptoms, making a better understanding of the etiologic mechanisms underlying NPS in AD critical to develop improved treatments.

**Method:**

To facilitate identification of genetic loci and mechanistic pathways underlying NPS in AD, we have initiated an effort (NIH: U01AG079850) to collate and harmonize all available NPS data in over 70 cohorts (>80,000 samples) of diverse ancestries with whole‐genome sequencing data from the Alzheimer’s Disease Sequencing Project (ADSP), and analyze these data to identify genetic loci and mechanistic pathways associated with NPS in AD.

**Result:**

A publicly available genomics resource for NPS in AD with extensive harmonized NPS phenotype data. Primary core analyses will (1) identify novel genetic risk factors associated with neuropsychiatric symptoms in AD, (2) characterize the shared genetic architecture of neuropsychiatric symptoms in AD and primary psychiatric disorders, and (3) assess the role of ancestry effects in the etiology of neuropsychiatric symptoms in AD.

**Conclusion:**

Expansion of the ADSP to harmonized and refined NPS phenotypes coupled with the proposed core analyses will lay the foundation to disentangle the molecular mechanisms underlying these detrimental symptoms in AD in diverse populations.

